# Implementation of BMI field charts for nutritional assessment in adult patients with tuberculosis in Karnataka

**DOI:** 10.5588/pha.25.0017

**Published:** 2025-09-03

**Authors:** M. Bhargava, K.M. Akshaya, M.N. Badarudeen, S.B. Nagaraja, A. Bhargava

**Affiliations:** ^1^ Department of Community Medicine, Yenepoya Medical College, Mangalore, India;; ^2^ Center for Nutrition Studies, Yenepoya (Deemed to be University), Mangalore, India;; ^3^ District TB Center, Mangalore, India;; ^4^ Department of Community Medicine, ESIC Medical College and PGIMSR, Bangalore, India;; ^5^ Department of Medicine, Kasturba Medical College Mangalore, Manipal Academy of Higher Education, Manipal, India;; ^6^ Department of Medicine, McGill University, Montreal, Canada.

**Keywords:** nutritional assessment, primary health centres, undernutrition

## Abstract

**BACKGROUND:**

We tested the operational feasibility of body mass index (BMI) field charts in nutritional assessment of adult patients with tuberculosis (PwTB), which obviate calculations and provide nutritional status based on BMI and the ideal weight (BMI = 21 kg/m^2^).

**METHODS:**

We trained primary health care providers (HCPs) in 39 primary health centres for nutritional assessment and classification and identifying the ideal weight using BMI field charts in PwTB. Using the descriptive statistics method, we analysed the collected data and reported the nutritional status in PwTB and the uptake of the field charts among the HCPs.

**RESULTS:**

The median (interquartile range [IQR]) weight and BMI were 44 kg (37.0, 50.0) and 16.9 kg/m^2^ (15.2, 18.9), respectively, in 214 PwTB, of which 146 (68.2%) patients had a BMI of <18.5 kg/m^2^. The HCPs documented the ideal weight in 155 (72.4%) patients, which was correct in 147 (94.8%) patients. The median (IQR) weight deficit to achieve the ideal weight was 10.4 kg (7.3, 12.8) in men and 11.9 kg (7.0, 17.9) in women. For a BMI of 18.5 kg/m^2^, the deficit was 6.4 kg (3.4, 8.5) in men and 11.3 kg (4.6, 13.6) in women.

**CONCLUSION:**

The magnitude and severity of undernutrition in adult PwTB in a well-performing district of Karnataka in South India were high. A single training session successfully improved nutritional assessment and BMI field chart usage among the primary HCPs.

Undernutrition is a serious comorbidity in patients with tuberculosis (PwTB).^1–3^ Globally, about 1 million PwTB suffer from undernutrition, and this comorbidity is much higher in high TB-burden countries such as India.^2,3^ The United Nations High-Level Meeting recognised undernutrition as a barrier to TB elimination and has committed to multisectoral action on undernutrition.^4^ Undernutrition is associated with severe TB and is a risk factor for death, adverse drug reactions, delayed sputum conversion, and prolonged transmission.^5–8^ In the absence of weight gain early during treatment, chances of TB recurrence also increase.^3^ The National TB Elimination Programme (NTEP) of India has undertaken several initiatives such as adapting the WHO guidelines for nutritional care and support of PwTB in 2017, implementing direct benefit transfer to bank accounts to support nutrition, and launching the Pradhan Mantri TB Mukt Bharat Abhiyan, where civil society and the corporate sector provide food baskets to PwTB.^9–11^

Public health tools that simplify nutrition screening at the start of treatment, support weight gain monitoring during treatment, and identify individuals who require additional nutritional support or referral for admission are essential in high TB-burden countries.^9,12^ The body mass index (BMI) chart remains an important public health tool due to its simplicity and well-established cut-offs for undernutrition. BMI field charts help the user classify the nutritional status and estimate the ideal weight without the manual calculation of BMI.^12^ Identifying severe undernutrition in PwTB and their timely referral can prevent mortality and morbidities.^13,14^

We conducted a study in primary health centres (PHCs) to estimate the burden of undernutrition among adult PwTB registered under the NTEP and to evaluate the operational feasibility of using BMI field charts for nutritional assessment in primary care.

## MATERIAL AND METHODS

### Study setting

This cross-sectional study was conducted in two tuberculosis units (TUs) located in Mangalore and Bantwala cities of the Dakshina Kannada district of Karnataka, South India. The NTEP is implemented through programme management units called TUs, with PHCs catering to approximately 30,000 patients each. These facilities have health care providers (HCPs) such as medical officers, staff nurses, and paramedics who provide preventive, promotive, and curative care. We included all 39 PHCs of these two TUs. Their TB services include screening, diagnosis, treatment initiation, supervision, follow-up, and maintaining programmatic documents in the form of TB cards for the NTEP.

### Participants, sampling, and sample size

We included all adult PwTB (≥18 years old) who were diagnosed and being treated in the 39 PHCs between January 2018 and June 2018 by using the complete enumeration method.

### Implementation process

After a baseline evaluation of the availability and functionality of weight- and height-measuring equipment, we conducted in-person training sessions of 30–45 min each for all HCPs in each PHC, which included discussing the importance of nutrition in PwTB, using BMI field charts, and a practice session.^15^ We provided an additional form to note the ideal weight identified from the field charts. The data in these forms (primary data) and the select information in the TB card (secondary data) were collected at the end of the study.

### BMI field charts and nutritional status classification

The BMI field charts for adults obviate complex exponential calculations to classify the nutritional status and provide weights against the adult heights in colour-coded tables in various BMI ranges.^12^ The WHO classification of adult nutritional status was used to categorise the nutritional status – normal: 18.5–24.99 kg/m^2^; overweight/obese: ≥25.0 kg/m^2^; mild undernutrition: 17.0–18.49 kg/m^2^; moderate undernutrition: 16.0–16.99 kg/m^2^; severe undernutrition: <16.0 kg/m^2^. An additional category of extremely severe undernutrition (BMI < 14 kg/m^2^) associated with a very high mortality risk is also available.

### Ideal weight and desirable weight

A column for ideal body weight enables HCPs to estimate the target weight to be achieved by a patient, corresponding to a BMI of 21 kg/m^2^ for a given adult height. The rationale for using 21 kg/m^2^ as an ideal BMI is based on the Malnutrition Universal Screening Tool, as well as the new definition of malnutrition in adults proposed by the European Society for Clinical Nutrition and Metabolism, where a BMI of <20 kg/m^2^ is considered low and not 18.5 kg/m^2^.^16,17^ PwTB often lose weight during follow-up after resuming work, so a target BMI of 18.5 kg/m^2^ may lead to categorising them as undernutrition. Operationally, the desirable body weight corresponded to a BMI of 18.5 kg/m^2^.

### Data sources and variables

Demographic characteristics, disease particulars, haemoglobin levels, if available, and anthropometric details at the baseline were obtained from the TB cards. The cards also have weight bands (25–39, 40–54, 55–70, and >70 kg) for appropriate dosing of anti-TB drugs. During the baseline evaluation of PwTB in the PHCs, it was noted that the weight bands were sometimes used to identify undernutrition, and the regular BMI calculation was often found challenging.^15^

### Measurements

We used the baseline weights and heights as measured under the programmatic conditions in the PHCs. The equipment varied from dial-type weighing machines to digital scales for weight and stadiometers, wall-mounted stature-meters, and even wall-markings in a few cases for height.^15^ The HCPs documented the ideal weight identified from the field chart in the additional form provided during the training.

### Statistics

Descriptive statistics for quantitative and categorical variables were presented using medians (interquartile range [IQR]) and percentages. Mann-Whitney U and χ^2^ tests were used to test for differences in medians and percentages. A *P* value of <0.05 was considered significant. The statistical analysis was performed with STATA version 17.0.

### Ethics

Ethics clearance for the study was obtained from the Institutional Ethics Committee (YUEC: 241/2017) of Yenepoya Medical College, Mangalore, and permission to conduct the study was obtained from district health authorities. We obtained written and informed consent from all participating HCPs.

## RESULTS

The socio-demographic and disease characteristics of 214 adult PwTB enrolled in the PHCs during the study period are given in Table 1: >80% had pulmonary TB (PTB), 75% had microbiologically confirmed TB, and more than two thirds were below the poverty line. The HIV status was documented in 99% of the TB cards, and 9 (4%) patients were reactive; 18% of the patients had coexisting diabetes documented in 210 cards. A history of alcohol and tobacco use was noted in 44/198 (22.2%) and 65/201 (32.3%) patients, respectively.

**TABLE 1.** Demographic and disease characteristics of adult patients with TB in the Dakshina Kannada district.

Characteristics of patients (*N* = 214)	Frequency	Percentage
Sex		
Males	136	63.6
Females	78	36.4
Socio-economic status		
Above poverty line	33	15.4
Below poverty line	154	72.0
Not entered	27	12.6
Disease site		
Pulmonary	173	80.8
Extra-pulmonary	41	19.2
Type of patient		
New	154	71.9
Recurrent	43	20.2
Treatment after failure/LTFU	17	7.9
Diagnosis		
Microbiological confirmation	166	77.6
Clinical diagnosis	48	22.4
Comorbidities and risk factors		
Diabetes	38/210	18.1
HIV	9/211	4.2
Alcohol use	44/198	22.2
Tobacco use	65/201	32.3

LTFU = loss to follow-up.

No weight or height measurement data were found to be missing. As shown in Table 2, nearly half of the men and women had a weight of <45 and 37 kg and a BMI of <17 and 16.6 kg/m^2^, respectively. The weights ranged from 30.5 to 71 kg in men and 25 to 69 kg in women, and the BMI range was 11.6–27.2 kg/m^2^ in men and 10.3–28.4 kg/m^2^ in women (data not shown). More than 80% of men were in the 25–39 kg and 40–54 kg weight bands, and >90% of women belonged to these weight bands. Almost half of the women had a baseline weight in the lowest weight band of 25–39 kg and a BMI of <16 kg/m^2^. Undernutrition was prevalent in more than two thirds of both men and women. Nearly a quarter of women had a BMI of <14 kg/m^2^. Of the 43 patients (20.1%) who were tested for anaemia, the median haemoglobin level was 10.8 g/dl, 36 (83.7%) had anaemia, and 25 (64%) had moderate anaemia (data not shown).

**TABLE 2.** Nutritional status and weight bands of adult patients with TB in Dakshina Kannada.

Nutritional parameters	Overall	Men	Women
Baseline weight, median (IQR), kg	44 (37.0, 50.0)	45 (40.7, 53.0)	37 (33.0, 45.0)**^A^**
Height, median (IQR), cm	158 (153, 165)	162 (153, 168)	153.8 (148, 157)
BMI, median (IQR), kg/m^2^	16.9 (15.2, 18.9)	17.0 (16.0, 18.9)	16.6 (13.9, 19.3)**^B^**
Weight bands, *n* (%)**^C^**			
25–39 kg	66 (30.8)	21 (15.4)	45 (57.7)**^D^**
≥40–54 kg	118 (55.1)	91 (66.9)	27 (34.6)
≥55–70 kg	28 (13.1)	22 (16.2)	6 (7.7)
>70 kg	2 (0.9)	2 (1.5)	0
BMI categories, *n* (%)			
<18.5 kg/m^2^	146 (68.2)	91 (66.9)	55 (70.5)
<14.0	28 (13.1)	9 (6.6)	19 (24.4)**^E^**
≥14.0–15.9	37 (17.3)	19 (14.0)	18 (23.1)
≥16.0–16.9	48 (22.4)	40 (29.4)	8 (10.3)
≥17.0–18.4	33 (15.4)	23 (16.9)	10 (12.8)
≥18.5 kg/m^2^	68 (31.8)	45 (33.1)	23 (29.5)
≥18.5–24.9	56 (26.2)	36 (26.5)	20 (25.6)
≥25.0	12 (5.6)	9 (6.6)	3 (3.9)

A *P* < 0.000005 (Mann-Whitney U test).

B *P* = 0.0057 (Mann-Whitney U test).

C Weight bands are for drug dosing as per the NTEP, India.

D *P* < 0.0005 (χ^2^ test).

E *P* < 0.0005 (χ^2^ test).

Table 3 presents median weights, BMI, and the proportion of low BMI stratified by sites of TB disease, socio-economic status, comorbidities, and behavioural risk factors. The prevalence of low BMI was around 50% in patients with diabetes and those with extra-pulmonary TB (EPTB) and around 75% in those with PTB and alcohol use. The median BMI was 16.7 kg/m^2^ in patients with tobacco use and 18.7 kg/m^2^ in patients with diabetes. A significant difference was observed in the median weight and BMI of patients with PTB and EPTB.

**TABLE 3.** Nutritional status in adult patients with TB with comorbidities and risk factors in Dakshina Kannada.

Comorbidity/risk factor	BMI < 18.5, *n*/*N* (%), kg/m^2^	Median weight (IQR)	*P* value**^A^**	Median BMI (IQR)	*P* value**^A^**
Disease site					
Pulmonary	125/173 (73.3)	42.0 (36.7, 49.0)	0.0001	16.8 (14.9, 18.7)	0.0001
Extra-pulmonary	21/41 (51.2)	49.0 (43.0, 56.0)		18.4 (16.8, 22.5)	
Socioeconomic status					
Below poverty line	110/153 (71.9)	43.0 (37.0, 49.0)	0.2758	16.8 (15.2, 18.7)	0.5964
Above poverty line	22/34 (64.7)	43.5 (39.0, 52.0)		17.0 (15.5, 20.0)	
Diabetes screened					
Diabetes	18/38 (47.4)	45.0 (40.0, 52.0)	0.1103	18.7 (16.8, 20.2)	0.0019
No diabetes	126/172 (73.3)	43.0 (37.0, 49.0)		16.8 (15.0, 18.7)	
Risk factors					
Alcohol use	35/44 (79.6)	44.0 (39.0, 47.0)	0.6683	16.8 (15.1, 17.5)	0.1361
No alcohol use	98/154 (63.6)	44.0 (37.0, 51.0)		17.3 (15.4, 19.0)	
Tobacco use	47/65 (72.3)	44.0 (37.0, 47.0)	0.6943	16.7 (15.2, 18.6)	0.2550
No tobacco use	90/136 (66.2)	43.0 (37.0, 50.5)		17.3 (15.3, 19.0)	

A Mann-Whitney U test.

Table 4 shows median weights, BMI, and the prevalence of undernutrition and severe undernutrition in the different weight bands. Nearly all PwTB (66/214) were underweight (as per BMI) in the lowest weight band (25–29 kg). In the case of the 40–54 kg weight category (118/214), undernutrition was seen in >70% of men and women, and severe undernutrition (BMI < 16 kg/m^2^) was observed in 12% of men in this category. For the 55–70 kg weight category, around 20% of men would still be classified as underweight based on their BMI status. Two patients in the weight category of >70 kg had normal BMI (data not shown).

**TABLE 4.** Nutritional status in adult patients with TB in the different weight bands provided by the NTEP, India.

Weight bands	Median weight (IQR)	Median BMI (IQR)	BMI < 18.5 kg/m^2^	BMI < 16.0 kg/m^2^
Weight band 25–39 kg				
Overall (*n* = 66)	35.0 (30.5, 36.7)	14.2 (13.6, 15.8)	65 (98.5%)	51 (77.3%)
Men (*n* = 21)	36.0 (35.0, 39.0)	15.0 (13.9, 15.8)	21 (100%)	16 (76.2%)
Women (*n* = 45)	33.0 (30.0, 36.0)	14.1 (12.8, 15.5)	43 (95.6%)	35 (77.8%)
Weight band 40–54 kg				
Overall (*n* = 118)	45.0 (42.0, 50.0)	17.5 (16.6, 18.9)	76 (64.4%)	13 (11.0%)
Men (*n* = 91)	46.0 (42.0, 50.0)	17.2 (16.4, 18.7)	65 (71.4%)	11 (12.1%)
Women (*n* = 27)	45.0 (43.0, 48.0)	18.8 (17.4, 19.6)	19 (70.4%)	2 (7.4%)
Weight band 55–70 kg				
Overall (*n* = 28)	60.5 (54.0, 65.0)	22.0 (18.9, 26.3)	5 (17.9%)	1 (3.6%)
Men (*n* = 22)	60.5 (54.0, 65.0)	21.1 (18.6, 26.7)	5 (22.7%)	1 (4.5%)
Women (*n* = 6)	64.0 (56.0, 69.0)	24.5 (22.7, 25.7)	0	0

The NTEP programme staff documented the ideal weight from the field charts in 155/214 (72.4%) patients and identified the correct target weight in 147/155 (94.8%) patients. The median (IQR) weight difference between the ideal body weight to reach a BMI of 21 kg/m^2^ and the weight at baseline in the patients who were underweight (146/214) was 10.4 kg (7.3, 12.8) in men and 11.9 kg (7.0, 17.9) in women. The median (IQR) weight difference between the weights corresponding to a BMI of 18.5 kg/m^2^ (desirable weight) was 6.4 kg (3.4, 8.5) in men and 11.3 kg (4.6, 13.6) in women.

## DISCUSSION

This is one of the first feasibility studies to understand the utility of BMI field charts from the NTEP’s Guidance Document on Nutritional Care and Support for PwTB in primary care.^9,12^ After a single training session, the HCPs recorded the weight and height in all PwTB (100%) with available resources, enabling the assessment of the prevalence and severity of undernutrition. The HCPs’ compliance with the usage guideline of the charts was encouraging, with a high concordance between the ideal weights in the charts and those recorded by the HCPs. While more than two thirds of the patients suffered from undernutrition, the prevalence and severity of undernutrition were higher in women, and nearly half of them had severe undernutrition.

The weight bands provided by the NTEP are for dosing; thus, the assessment of nutritional status based on the weight bands can be fallacious. More than 80% of men and 90% of women belonged to the lower two weight bands (25–39 and 40–54 kg). Two thirds of men and one third of women were in the 40–54 kg weight band, and more than half of the women were in the lowest weight band of 25–39.9 kg.

The weight and BMI of the patients did not differ when the nutritional status was stratified by socio-economic status, alcohol use, or tobacco use. Patients with EPTB and diabetes had higher BMI; however, nearly half of the patients with diabetes were underweight. Of concern is undernutrition that was high across the categories of disease, socio-economic status, comorbidities, and risk factors in PwTB in a district that ranks first in literacy and second in per capita income and human development index in Karnataka. Recent studies in India have reported the prevalence of undernutrition in PwTB from 54.9% to 82%.^3,13^ The median weights observed in this survey, 42 kg for men and 38 kg for women, are similar to those reported in the NTEP in 2016^18^ and to the mean weights in the patient cohort of the RATIONS trial in Jharkhand (43 kg in men and 36 kg in women).^13^ In a multicentric study and a systematic review of malnutrition in tribal PwTB, Madhya Pradesh, Odisha, Chhattisgarh, and Bengal give a similar picture of undernutrition in TB.^19–23^ The presence of undernutrition in a substantial proportion of PwTB with coexisting diabetes has been noted in studies from India^24^ and other countries.^25,26^ The interplay of poor diabetes control and TB and a phenotype of low-body-weight type 2 diabetes mellitus in low- and middle-income countries must be kept in mind while managing these patients.

This study demonstrated the feasibility of nutritional assessment of all PwTB when primary HCPs are provided with appropriate tools, training, and supportive supervision. The BMI charts provide care providers a target weight, which is otherwise challenging to arrive at in a busy primary care setting. Nutritional classification is of prognostic value and triage of PwTB and is used in decision making for nutritional support. Moderate to severe undernutrition and poor weight gain are strong predictors of unfavourable outcomes, including a two-to-four-fold higher risk of mortality during and after treatment, a higher risk of drug toxicity, and recurrent TB.^1,3,27^ Severe undernutrition and extremely severe undernutrition are a threat to patients’ lives and are surrogates for disease severity. The guidance document proposes BMI < 14 or 16 kg/m^2^ with oedema, poor vital signs, and other medical conditions as triage criteria for referral and inpatient care.^9^ It has been successfully implemented in Tamil Nadu in the differentiated care model and the RATIONS trial.^13,28^ Recent evidence suggests that even a 5% weight gain in the intensive phase with the provision of nutritional support reduces the hazard of death by >60%.^13^ In the absence of nutritional support, patients may experience a static or declining nutritional status in the first 2 months, with a five-fold higher risk of death.^13^

This study provides actionable information on nutritional status at diagnosis and follow-up based on weight and height measurements without calculating BMI. The study highlights that arbitrary interpretation of nutritional status, including that based on the weight bands, is likely to be fallacious and leads to underestimation of undernutrition and its severity.^14,15,29^

The information obtained from the field charts may also help determine nutritional support packages for patients in different parts of the country. On the basis of the results of this study, underweight men with TB may need to gain 6.4 kg to attain a BMI of 18.5 kg/m^2^ and 10.4 kg to attain a BMI of 21 kg/m^2^. The corresponding weight gain for women would be close to 11 kg. A median weight gain of 4.6 kg was observed with a 10-kg food basket over 6 months in the RATIONS trial.^13^ Achieving a higher weight gain, reaching a minimum BMI of 18.5 kg/m^2^ or an optimal BMI of 21 kg/m^2^, may require nutritional support beyond the 6-month treatment period, given that there are physiological limits to weight gain (∼7,500 kcal needed for 1 kg weight gain). Without the recording and reporting of BMI in the NTEP, it would be impossible to determine the extent of nutritional support, allocate resources effectively, conduct proper monitoring, and assess the long-term impact of any policy intervention. The BMI charts can help assess adult PwTB at baseline, support the provision of nutritional support and counselling, monitor weight gain during treatment, assess and report nutritional status at the end of treatment, assess for possible failure and relapse, and link patients to post-treatment welfare schemes where appropriate.^30^

Significant limitations of the study include a limited geographic region (single district) and anthropometry, which was done under programmatic conditions in a non-standardised manner. However, this study provides a pointer towards the possible use of the BMI field charts, which can support monitoring and evaluation of nutrition support initiatives by the NTEP.

## CONCLUSION

The prevalence and severity of undernutrition in PwTB in a well-performing district of Karnataka in South India were high, indicating the need for nutritional assessment and support for patients in this region. A single training session focused on assessing nutritional status using BMI field charts enabled the HCPs to completely record of the height and weight of all patients and thus motivated most care providers to identify a target weight to be achieved. The NTEP should consider integrating BMI field charts in nutritional assessment and differentiated care toolkits to improve patient outcomes for this widely prevalent, critical, but reversible comorbidity of TB in India.
